# Association of the Modified HALP Score with Disease Complexity in Deep Neck Infections: A Dual-Center Retrospective Study

**DOI:** 10.3390/diagnostics16162604

**Published:** 2026-08-17

**Authors:** Serkan Serifler, Serdal Celik, Berina Slipcevic, Fatih Gul, Burak Celik, Kadir Sinasi Bulut, Kazim Bozdemir, Mahmut Tayyar Kalcioglu

**Affiliations:** 1 Department of Otolaryngology, Head and Neck Surgery, School of Medicine, Ankara Yildirim Beyazit University, Ankara 06000, Türkiye; burakcelik70@gmail.com (B.C.); kadirsinasibulut@gmail.com (K.S.B.); kazimbozdemir@gmail.com (K.B.); 2 Department of Otolaryngology, Head and Neck Surgery, School of Medicine, Istanbul Medeniyet University, Istanbul 34000, Türkiye; serdal.celik77@hotmail.com (S.C.); berinaslipcevic@gmail.com (B.S.); mtkalcioglu@hotmail.com (M.T.K.); 3 Department of Otolaryngology, Head and Neck Surgery, School of Medicine, Lokman Hekim University, Ankara 06000, Türkiye; drfatihgul@gmail.com

**Keywords:** deep neck infections, inflammation, modified HALP score, prognostic biomarkers

## Abstract

**Background/Objectives**: To evaluate the association between the modified hemoglobin–albumin–lymphocyte–platelet (mHALP) score and disease complexity in patients hospitalized with deep neck infections (DNIs) and to compare performance with HALP and individual laboratory markers. **Methods**: This dual-center retrospective study included 367 adults hospitalized with DNIs between December 2021 and September 2025. Patients were classified as having complicated or non-complicated disease. The mHALP score was calculated from admission laboratory values and incorporated C-reactive protein. ROC curve and multivariable logistic regression analyses were performed. The primary model evaluated natural-log-transformed, standardized mHALP after adjustment for age, maximum abscess diameter, and study center. **Results**: Fifty-nine patients (16.1%) had complicated DNIs. Median mHALP was lower in the complicated group than in the non-complicated group (0.0137 vs. 0.0486; *p* < 0.001). mHALP showed moderate discrimination (AUC = 0.756, 95% CI 0.677–0.834) and performed better than CRP alone (AUC difference = 0.077; *p* < 0.001), but not better than HALP, albumin, or lymphocyte count. An exploratory cut-off of ≤0.0302 yielded 74.6% sensitivity and 71.8% specificity. In the primary multivariable model, each one-standard-deviation increase in log-transformed mHALP was associated with lower odds of complicated disease (adjusted OR = 0.592, 95% CI 0.397–0.884; *p* = 0.010). Adding mHALP to the clinical model increased the AUC from 0.858 to 0.864, whereas adding HALP increased it to 0.894. **Conclusions**: Lower mHALP was independently associated with complicated DNIs, but its incremental value beyond clinical variables was modest, and it did not outperform HALP. mHALP may be considered an adjunctive, rather than stand-alone, marker, pending external validation.

## 1. Introduction

Despite advances in antibiotic therapy, imaging, and surgical management, deep neck infections (DNIs) remain potentially life-threatening conditions. If they are not recognized and treated promptly, they can progress rapidly and lead to serious complications, including airway obstruction, mediastinitis, sepsis, and death [1,2,3]. Therefore, early identification of patients at risk of a complicated clinical course is essential for guiding treatment decisions and improving clinical outcomes [4].

The clinical presentation of DNIs is often heterogeneous. Although conventional laboratory markers such as leukocyte count and C-reactive protein (CRP) are widely used in routine practice, they have limited ability to predict disease severity when interpreted in isolation [5,6]. For this reason, there has been an increasing focus on composite inflammatory and nutritional indices that consider multiple aspects of the host response, such as systemic inflammation, immune status, and nutritional reserve [7,8].

The hemoglobin, albumin, lymphocyte, and platelet (HALP) score is one such index that has been investigated as a prognostic marker in oncological, inflammatory, and infectious diseases [9,10,11]. By integrating hematological, nutritional, and immune-related parameters, the HALP score may provide information that goes beyond that offered by individual laboratory tests [12]. HALP has also been evaluated in acute infectious conditions, including sepsis, although its prognostic performance has varied across study populations [13,14].

In cases of acute infection and emergency surgical conditions, including appendicitis and selected gastrointestinal infections, HALP-based indices have demonstrated the ability to distinguish between complicated and non-complicated disease, as well as identify patients at a higher risk of adverse outcomes [15,16]. However, data on the modified HALP score in patients with DNI remains limited. Due to the rapid clinical progression and potentially severe complications associated with DNIs, having a simple, readily available marker to support early risk stratification could be clinically useful.

Previous studies have evaluated the HALP score in patients with DNIs, reporting associations with inflammatory burden and clinical severity [17]. However, it remains unclear whether incorporating CRP into the HALP formula improves discrimination compared with HALP or its individual laboratory components. Therefore, the present study evaluated the association between a CRP-adjusted modified HALP score and complicated DNIs. We also compared its discriminatory performance with that of HALP, CRP, albumin, and lymphocyte count and examined whether mHALP provided additional information beyond a clinical model including age, maximum abscess diameter, and study center.

## 2. Materials and Methods

### 2.1. Ethical Approval

This study was conducted in accordance with the Declaration of Helsinki and approved by the Clinical Research Ethics Committee of Göztepe Prof. Dr. Süleyman Yalçın City Hospital (approval number/date: 2026-0299/6 May 2026). The requirement for informed consent was waived due to the retrospective design of the study. All patient data were de-identified before analysis and handled in accordance with institutional data protection requirements.

### 2.2. Study Design and Patient Selection

This dual-center retrospective observational study included patients aged 18–65 years who presented to the emergency departments of two tertiary referral centers with DNIs and were subsequently hospitalized between December 2021 and September 2025. The age range of 18–65 years was defined a priori as part of the study eligibility criteria and applied consistently at both centers. The participating institutions were Ankara Bilkent City Hospital, located in Ankara, Türkiye, as Center 1, and Göztepe Prof. Dr. Süleyman Yalçın City Hospital, located in İstanbul, Türkiye, as Center 2. Both institutions are tertiary referral hospitals providing emergency, otolaryngology, radiology, intensive care, and operative services for patients with DNIs. A DNI diagnosis was made based on clinical findings and confirmed by ultrasonography and/or contrast-enhanced computed tomography showing abscess formation. Both centers applied identical eligibility criteria and used a standardized data extraction protocol.

Patients outside the specified age range, those who declined hospitalization, and those with incomplete medical records or missing laboratory parameters required for modified HALP calculation were excluded. Only patients with complete data for all variables included in the primary analyses were retained, and no missing values were imputed. No a priori sample size calculation was performed. All eligible patients treated during the prespecified study period were included in the analysis.

### 2.3. Patient Categorization

Patients were classified using an investigator-defined composite outcome established before data analysis and applied consistently at both centers. The criteria were not adopted from a previously validated classification system.

The non-complicated group (Group 1) included patients who did not meet any of the criteria defined for complicated disease and had a favorable clinical course with conservative management or simple bedside drainage.

The complicated group (Group 2) included patients who met one or more of the following criteria: (1) multispace involvement; (2) admission to the intensive care unit; (3) surgical drainage under general anesthesia in the operating theater (excluding bedside incision and drainage procedures); or (4) development of mediastinitis.

These endpoints were grouped to capture complementary aspects of disease complexity, including anatomical spread, physiological deterioration, and the need for escalation to invasive treatment.

Treatment decisions, including intensive care unit admission and surgical drainage under general anesthesia, were made by the attending clinical teams according to the patient’s clinical condition, airway status, radiological findings, and response to initial treatment. Both centers followed similar general principles of DNI management; however, no unified written protocol was in place during the study period. Study center was therefore included in the multivariable models to account for potential institutional differences in management.

### 2.4. Data Collection

Data were collected retrospectively from medical records using a standardized data extraction form. The following variables were recorded: demographic information (age and sex), abscess characteristics (anatomical location and maximum diameter), treatment modality, drainage status, complete blood count parameters (hemoglobin, white blood cell count, neutrophils, lymphocytes, monocytes, and platelets), serum albumin and C-reactive protein (CRP) levels at hospital admission, as well as length of hospital stay.

All laboratory values were obtained at the time of initial hospital admission, prior to any surgical intervention. Complete blood count parameters were measured using fully automated Sysmex hematology analyzers at both centers. Serum C-reactive protein and albumin concentrations were measured according to the manufacturers’ instructions using the Atellica CH analyzer (Siemens Healthineers, Erlangen, Germany) at Center 1 and the Cobas c 702 analyzer (Roche Diagnostics, Basel, Switzerland) at Center 2. Although the clinical chemistry analyzer platforms were not identical, both laboratories used standardized analytical procedures and common reporting units. The same variable definitions and data extraction procedures were applied at both centers to minimize inter-center variability.

### 2.5. HALP and Modified HALP Score Calculation

The HALP score was calculated using the following formula:

HALP = [hemoglobin (g/dL) × albumin (g/L) × lymphocyte count (×10^9^/L)]/platelet count (×10^9^/L)

The modified HALP score was calculated by incorporating CRP into the denominator of the HALP formula:

mHALP = [hemoglobin (g/dL) × albumin (g/L) × lymphocyte count (×10^9^/L)]/[platelet count (×10^9^/L) × CRP (mg/L)]

Thus, mHALP corresponded to the HALP score divided by CRP. Neither HALP nor mHALP was routinely calculated or used for clinical decision-making at either center during the study period. For the purposes of this study, both scores were retrospectively derived from routinely obtained admission laboratory measurements. As DNIs represent an acute bacterial inflammatory condition, CRP was incorporated to account for the systemic inflammatory burden at presentation. This modification was intended as a context-specific adaptation of the HALP score rather than as an established prognostic index.

### 2.6. Statistical Analysis

Categorical variables were summarized as frequencies and percentages. As continuous variables were not normally distributed, they were expressed as the median and interquartile range (IQR). The Mann–Whitney U test was used for continuous variables, and the chi-squared or Fisher’s exact test was used for categorical variables, as appropriate.

Receiver operating characteristic (ROC) curve analysis was performed to evaluate the ability of HALP, CRP, albumin, lymphocyte count, and mHALP to distinguish complicated from non-complicated DNIs. Area under the curve (AUC) was reported with 95% confidence intervals. Optimal cut-off values were identified using the Youden index, with corresponding sensitivity and specificity estimates. Pairwise comparisons between the mHALP ROC curve and the curves of the other biomarkers were performed using the DeLong method for correlated ROC curves.

The primary multivariable analysis was performed using binary logistic regression, with complicated DNIs as the dependent variable. Because of its skewed distribution, mHALP was natural-log-transformed and standardized. Age, maximum abscess diameter, study center, and standardized log-transformed mHALP were entered simultaneously into the model. Center 1 was used as the reference category. Results were reported as adjusted odds ratios (ORs) with 95% CIs.

To assess the incremental value of the biomarkers, a clinical reference model was constructed, incorporating age, maximum abscess diameter, and study center. HALP, CRP, albumin, lymphocyte count, and mHALP were then added separately to this model. An additional model included both HALP and CRP. HALP, CRP, lymphocyte count, and mHALP were natural-log-transformed and standardized, whereas albumin was standardized without transformation. Model performance was assessed using the AUC, Akaike information criterion, Brier score, and likelihood-ratio tests. Changes in AUC relative to the clinical reference model were assessed using the DeLong method for correlated ROC curves.

Internal validation of the primary clinical plus mHALP model was performed using 2000 bootstrap resamples. In each resample, the prespecified model was refitted, and its performance was evaluated in both the bootstrap sample and the original cohort. The mean difference in performance was used to estimate optimism and obtain optimism-corrected AUC and Brier score values. A bootstrap-corrected calibration slope was also calculated.

The ROC-derived mHALP cut-off was evaluated in a secondary exploratory analysis. Crude and adjusted ORs were calculated, with adjustment for age, maximum abscess diameter, and study center. Because the cut-off was derived and evaluated in the same cohort, this analysis was considered exploratory.

A *p*-value < 0.05 was considered statistically significant. All analyses except the pairwise DeLong comparisons and bootstrap internal validation were performed using IBM SPSS Statistics for Windows, version 32.0 (IBM Corp., Armonk, NY, USA). The DeLong comparisons and bootstrap internal validation were performed using Python version 3.13-based prespecified analysis scripts.

## 3. Results

### 3.1. Patient Characteristics

A total of 367 patients were included in the final analysis. Of these, 292 patients (79.6%) were treated at Center 1 and 75 (20.4%) at Center 2. Based on predefined clinical criteria, 308 patients (83.9%) were classified as having non-complicated DNIs, whereas 59 patients (16.1%) were categorized as having complicated disease.

The baseline demographic, clinical, and laboratory characteristics of the study population are summarized in Table 1. Patients with complicated DNIs were older than those with non-complicated disease [median age, 38.0 years (IQR, 28.0–50.5) vs. 32.5 years (IQR, 26.0–42.0); *p* = 0.023]. Sex distribution did not differ significantly between the groups.

Patients with complicated DNIs had significantly lower hemoglobin concentrations [13.6 g/dL (IQR, 12.1–14.6) vs. 14.6 g/dL (IQR, 13.3–15.5); *p* = 0.001], serum albumin levels [41.0 g/L (IQR, 37.0–44.5) vs. 45.0 g/L (IQR, 43.0–47.0); *p* < 0.001], and lymphocyte counts [1.29 ×10^9^/L (IQR, 0.91–1.78) vs. 1.85 ×10^9^/L (IQR, 1.41–2.33); *p* < 0.001]. Platelet counts were numerically higher in the complicated group, although the difference did not reach statistical significance (*p* = 0.068).

CRP concentrations were significantly higher in patients with complicated disease [160.0 mg/L (IQR, 92.8–219.3) vs. 92.8 mg/L (IQR, 49.2–155.4); *p* < 0.001]. Both HALP and mHALP scores were significantly lower in the complicated group. The median HALP score was 2.282 (IQR, 1.569–3.421) in patients with complicated disease and 4.221 (IQR, 3.054–5.673) in those with non-complicated disease (*p* < 0.001). Corresponding median mHALP scores were 0.0137 (IQR, 0.0067–0.0335) and 0.0486 (IQR, 0.0279–0.0907), respectively (*p* < 0.001). The maximum abscess diameter was also substantially greater in the complicated group [45.0 mm (IQR, 30.0–66.0) vs. 21.0 mm (IQR, 16.0–28.0); *p* < 0.001] (Table 1).

### 3.2. Comparative Discriminatory Performance of the Biomarkers

**Figure 1:**
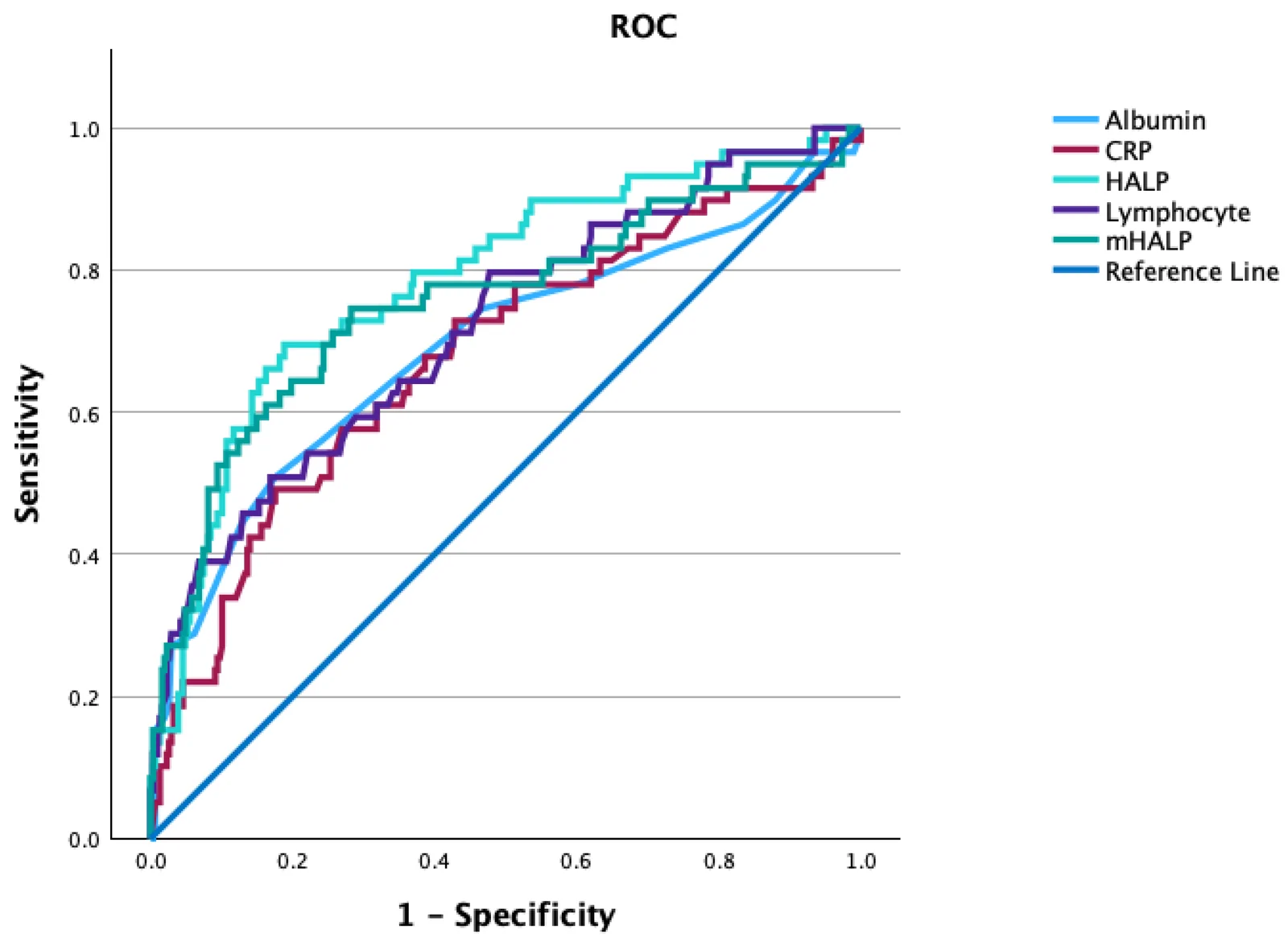
Comparative receiver operating characteristic curves of HALP, CRP, albumin, lymphocyte count, and mHALP for discriminating complicated from non-complicated deep neck infections.

Comparative receiver operating characteristic analyses for HALP, CRP, albumin, lymphocyte count, and mHALP are presented in Table 2 and Figure 1. All five biomarkers demonstrated statistically significant discrimination between complicated and non-complicated DNIs (all *p* < 0.001).

HALP had the highest AUC among the individual biomarkers, with an AUC of 0.786 (95% CI, 0.718–0.854). The optimal HALP cut-off was ≤2.7406, providing 69.5% sensitivity and 81.2% specificity. The mHALP score demonstrated moderate discrimination, with an AUC of 0.756 (95% CI, 0.677–0.834). A Youden-derived mHALP cut-off of ≤0.0302 yielded 74.6% sensitivity and 71.8% specificity.

The AUC values for lymphocyte count, albumin, and CRP were 0.719 (95% CI, 0.643–0.795), 0.692 (95% CI, 0.607–0.777), and 0.679 (95% CI, 0.598–0.760), respectively. The corresponding optimal cut-off values were ≤1.29 ×10^9^/L for lymphocyte count, ≤41 g/L for albumin, and ≥175 mg/L for CRP (Table 2 and Figure 1).

Pairwise DeLong comparisons are shown in Table 3. The discriminatory performance of mHALP was significantly better than that of CRP alone, with an AUC difference of 0.077 (95% CI, 0.040–0.113; *p* < 0.001). However, mHALP did not significantly outperform HALP (AUC difference, −0.030; 95% CI, −0.092 to 0.032; *p* = 0.339), albumin (AUC difference, 0.064; 95% CI, −0.020 to 0.148; *p* = 0.137), or lymphocyte count (AUC difference, 0.037; 95% CI, −0.039 to 0.113; *p* = 0.343) (Table 3).

### 3.3. Primary Multivariable Analysis

The primary multivariable logistic regression analysis evaluated mHALP as a continuous, natural-log-transformed, and standardized variable. The model included age, maximum abscess diameter, study center, and log-transformed mHALP.

Maximum abscess diameter was independently associated with complicated disease. Each 1 mm increase in abscess diameter was associated with a 9.0% increase in the odds of complicated DNI (adjusted OR = 1.090, 95% CI, 1.062–1.119; *p* < 0.001). Age was also associated with complicated disease, although the magnitude of the association was modest (adjusted OR = 1.031 per year, 95% CI, 1.000–1.063; *p* = 0.047). Study center was not independently associated with the outcome (Center 2 vs. Center 1: adjusted OR = 1.249, 95% CI, 0.475–3.287; *p* = 0.652).

Higher log-transformed mHALP values were independently associated with lower odds of complicated disease. Each one-standard-deviation increase in log-transformed mHALP was associated with an approximately 41% reduction in the odds of complicated DNI (adjusted OR = 0.592, 95% CI, 0.397–0.884; *p* = 0.010) (Table 4).

### 3.4. Incremental Performance of Biomarker-Enhanced Clinical Models

The performance of the clinical model and the biomarker-enhanced models is summarized in Table 5. The clinical model, which included age, maximum abscess diameter, and study center, had an AUC of 0.858, an AIC of 218.25, and a Brier score of 0.0829.

Adding HALP to the clinical model was associated with a significant improvement in model fit according to the likelihood-ratio test (*p* < 0.001). In this model, HALP remained independently associated with complicated disease (adjusted OR = 0.458 per one-standard-deviation increase, 95% CI, 0.317–0.661; *p* < 0.001). The resulting model had an AUC of 0.894, an AIC of 201.37, and a Brier score of 0.0769.

The model containing lymphocyte count also demonstrated a significant improvement over the clinical model according to the likelihood-ratio test (*p* < 0.001). Lymphocyte count was independently associated with complicated disease (adjusted OR = 0.449, 95% CI, 0.302–0.668; *p* < 0.001), and the model had an AUC of 0.883 and a Brier score of 0.0749.

Adding mHALP to the clinical model significantly improved model fit according to the likelihood-ratio test (*p* = 0.009), and mHALP remained independently associated with the outcome (adjusted OR = 0.592, 95% CI, 0.397–0.884; *p* = 0.010). However, the increase in model discrimination was limited, with the AUC increasing from 0.858 for the clinical model to 0.864 for the clinical plus mHALP model. The corresponding AIC and Brier score were 213.36 and 0.0794, respectively. When model AUC was compared directly with that of the clinical model, the increase after adding mHALP was small and not statistically significant (ΔAUC = 0.006; DeLong *p* = 0.656), whereas adding HALP produced a larger and statistically significant increase (ΔAUC = 0.036; DeLong *p* = 0.042).

Neither CRP nor albumin was independently associated with complicated disease after adjustment for the clinical variables (CRP: adjusted OR = 1.054, 95% CI, 0.719–1.546; *p* = 0.786; albumin: adjusted OR = 0.801, 95% CI, 0.576–1.115; *p* = 0.189). Their addition did not significantly improve model fit according to the likelihood-ratio tests.

In the model containing both HALP and CRP, HALP remained independently associated with complicated disease (adjusted OR = 0.444, 95% CI, 0.304–0.650; *p* < 0.001), whereas CRP did not (adjusted OR = 0.876, 95% CI, 0.585–1.313; *p* = 0.522). This combined model had an AUC of 0.896, an AIC of 202.97, and a Brier score of 0.0771 (Table 5).

Bootstrap internal validation of the primary clinical plus mHALP model showed a mean AUC optimism of 0.013. The apparent AUC of 0.864 was reduced to an optimism-corrected AUC of 0.851. The optimism-corrected Brier score was 0.0836, compared with an apparent value of 0.0794, and the bootstrap-corrected calibration slope was 0.939. These findings indicated limited overfitting of the primary model.

### 3.5. Secondary Exploratory Cut-Off Analysis

In a secondary exploratory analysis, patients were categorized according to the Youden-derived mHALP cut-off of ≤0.0302. Forty-four of the 59 patients with complicated DNIs and 87 of the 308 patients with non-complicated DNIs had mHALP values at or below this threshold. The cut-off provided 74.6% sensitivity and 71.8% specificity.

Patients with mHALP values ≤0.0302 had substantially higher crude odds of complicated disease than those with higher values (crude OR = 7.451, 95% CI, 3.943–14.080; *p* < 0.001). The association remained significant after adjustment for age, maximum abscess diameter, and study center (adjusted OR = 3.231, 95% CI, 1.491–7.002; *p* = 0.003) (Table S1). Because this threshold was derived and evaluated in the same cohort, these results should be regarded as exploratory.

## 4. Discussion

In this dual-center cohort, mHALP values were significantly lower in patients with complicated DNIs than in those with non-complicated disease. mHALP showed moderate discrimination and performed better than CRP alone, but it did not significantly outperform HALP, albumin, or lymphocyte count. When analyzed as a continuous variable, log-transformed mHALP remained independently associated with complicated disease after adjustment for age, maximum abscess diameter, and study center. However, its incremental contribution to the clinical model was modest, whereas HALP produced a greater improvement in overall model performance. These findings support an association between lower mHALP and disease complexity, but do not indicate that the CRP-adjusted formulation is superior to HALP.

HALP-based indices combine hematological, nutritional, and immunological parameters into a single composite score. Hemoglobin reflects anemia and inflammatory burden, albumin represents nutritional reserves and functions as a negative acute-phase reactant, lymphocyte count reflects immune competence, and platelet count is closely linked to inflammatory and thrombotic pathways [9,12]. While the original HALP score was introduced in oncology, subsequent studies have evaluated HALP and HALP-derived indices across a broad range of malignant and inflammatory conditions. This suggests that these scores may reflect overall host vulnerability rather than disease-specific mechanisms [10,11].

In the present study, CRP was incorporated into the HALP framework because DNIs represent an acute bacterial inflammatory condition. Unlike oncological or chronic disease settings, acute infections are characterized by rapid fluctuations in systemic inflammatory markers. Incorporating CRP into the denominator was intended to account for the intensity of inflammation at presentation and adapt the HALP concept to the clinical context of DNIs. The modified score was therefore examined as a context-specific adaptation of HALP rather than as an established prognostic index. Nevertheless, the present findings suggest that incorporating CRP into the formula did not improve discrimination over HALP and provided only limited additional information when added to the clinical model.

The prognostic performance of HALP and HALP-derived indices appears to vary across clinical settings. HALP has shown variable prognostic performance in patients with sepsis [13,14], a clinically heterogeneous condition in which organ dysfunction and overall illness severity are commonly assessed using multidimensional scoring systems such as SOFA and APACHE II [18,19]. In contrast, more localized inflammatory conditions, including pneumonia and selected gastrointestinal infections, have shown more consistent associations between HALP-based indices and clinical outcomes [15,16,20]. DNI occupies an intermediate position within this spectrum. Although it is typically a localized infection, it may progress rapidly and result in serious complications, such as airway compromise, mediastinitis, or sepsis [1,2,21]. Our findings are consistent with this variability and suggest that the relative contribution of the individual components may depend on the clinical setting and the outcome being assessed. Other inflammatory and nutritional indices, including the neutrophil-to-lymphocyte ratio, platelet-to-lymphocyte ratio, systemic immune-inflammation index, and prognostic nutritional index, have also been evaluated in patients with DNIs [22,23]. Iwata et al. reported that admission prognostic nutritional index was associated with prolonged hospitalization in patients with severe odontogenic DNIs [22]. In a retrospective study of 163 adults, Treviño-Gonzalez et al. found significantly higher NLR, PLR, and systemic immune-inflammation index values in patients who developed complications and reported that the systemic immune-inflammation index had a higher positive predictive value than NLR, PLR, and CRP in their cohort [23]. Although the outcomes and definitions used in these studies differed from ours, their findings support the potential role of composite inflammatory and nutritional indices in DNI risk assessment. We did not directly compare mHALP with these measures; therefore, future studies should determine whether mHALP provides additional information beyond established indices.

In the primary multivariable analysis, maximum abscess diameter showed the strongest association with complicated disease. Each 1 mm increase in abscess diameter was associated with a 9% increase in the odds of a complicated course. Age showed a weaker association, whereas study center was not independently associated with the outcome. After adjustment for these factors, each one-standard-deviation increase in log-transformed mHALP was associated with approximately 41% lower odds of complicated DNI. This association indicates that mHALP reflects information not fully represented by age, abscess size, or study center.

The model comparisons, however, provide a more cautious view of its clinical contribution. The clinical model already showed good discrimination, with an AUC of 0.858. Adding mHALP improved model fit but only increased the AUC to 0.864. In contrast, adding HALP increased the AUC to 0.894 and resulted in more favorable AIC and Brier score values. Lymphocyte count also improved model fit, whereas CRP and albumin did not. Moreover, when HALP and CRP were entered together, HALP remained independently associated with complicated disease, but CRP did not. Taken together, these findings suggest that the association observed for mHALP may be driven largely by the information already contained in the HALP components, with limited additional benefit from dividing the score by CRP.

Because mHALP is derived from routine admission laboratory tests, it offers a simple way to integrate inflammatory, nutritional, and hematological information. However, its modest incremental performance and lack of superiority over HALP argue against using mHALP as a stand-alone decision tool. Based on the present data, an mHALP value below the proposed threshold should not, by itself, prompt additional imaging, earlier surgery, ICU admission, or intensified monitoring. Such decisions should continue to be based on the overall clinical and radiological assessment. The ROC-derived threshold of approximately 0.03 should therefore be regarded as exploratory rather than a validated treatment or triage threshold.

Several limitations should be acknowledged. First, the retrospective design is subject to residual confounding and depends on the completeness of medical records. Although this was a dual-center study, the contribution of the two centers was unequal, and ICU admission and operative drainage were determined by the treating teams rather than by a shared written protocol. Center-specific differences in indications for ICU admission and operative drainage were not formally assessed. Because these management decisions formed part of the composite outcome, differences in institutional practice or clinician judgment may have influenced patient classification despite adjustment for study center. Information on smoking status, prior antibiotic use, symptom duration, immunosuppression, diabetes mellitus, and several comorbidities was not consistently available at both centers and therefore could not be included in the multivariable analyses. Although the anatomical space involved was recorded, the etiological source of infection, such as odontogenic or tonsillar origin, was not consistently documented. The limited number of complicated cases also restricted the number of covariates that could be included without increasing the risk of model overfitting. Because the study was restricted to adults aged 18–65 years, the findings may not be generalizable to patients older than 65 years. Although bootstrap internal validation suggested limited optimism in the performance of the primary model, this procedure does not replace external validation. The mHALP cut-off was derived and evaluated in the same cohort and should therefore remain exploratory until confirmed in an independent population. Moreover, mHALP did not outperform HALP or substantially improve discrimination beyond the clinical model. Finally, only laboratory values obtained at admission were assessed. Prospective studies with external validation, standardized management criteria, and adjustment for relevant comorbidities are needed.

## 5. Conclusions

In this dual-center cohort, lower mHALP values were associated with complicated DNIs and remained independently associated with the outcome when analyzed as a continuous variable after adjustment for age, maximum abscess diameter, and study center. mHALP showed moderate discriminatory performance and performed better than CRP alone; however, it did not outperform HALP and provided only a modest improvement beyond the clinical model. These findings suggest that mHALP may be considered an adjunctive marker rather than a stand-alone tool for clinical decision-making. The ROC-derived threshold should be regarded as exploratory and requires validation in independent prospective cohorts.

## Figures and Tables

**Table 1:** Comparison of demographic, clinical, and laboratory characteristics between patients with non-complicated and complicated deep neck infections.

Variable	Non-Complicated DNI (*n* = 308)	Complicated DNI (*n* = 59)	*p*-Value
Age, years, median (IQR)	32.5 (26.0–42.0)	38.0 (28.0–50.5)	0.023
Sex, n (%)			0.412
Male	205 (66.6)	36 (61.0)	
Female	103 (33.4)	23 (39.0)	
Study center, n (%)			0.281
Center 1	242 (78.6)	50 (84.7)	
Center 2	66 (21.4)	9 (15.3)	
Hemoglobin, g/dL, median (IQR)	14.6 (13.3–15.5)	13.6 (12.1–14.6)	0.001
Albumin, g/L, median (IQR)	45.0 (43.0–47.0)	41.0 (37.0–44.5)	<0.001
Lymphocyte count, ×10^9^/L, median (IQR)	1.85 (1.41–2.33)	1.29 (0.91–1.78)	<0.001
Platelet count, ×10^9^/L, median (IQR)	278.0 (235.8–332.3)	295.0 (248.5–371.0)	0.068
CRP, mg/L, median (IQR)	92.8 (49.2–155.4)	160.0 (92.8–219.3)	<0.001
HALP score, median (IQR)	4.221 (3.054–5.673)	2.282 (1.569–3.421)	<0.001
mHALP score, median (IQR)	0.0486 (0.0279–0.0907)	0.0137 (0.0067–0.0335)	<0.001
Maximum abscess diameter, mm, median (IQR)	21.0 (16.0–28.0)	45.0 (30.0–66.0)	<0.001

Footnote: Data are presented as median (interquartile range) or number (percentage), as appropriate. Continuous variables were compared using the Mann–Whitney U test, and categorical variables were compared using the chi-squared or Fisher’s exact test, as appropriate. CRP, C-reactive protein; DNI, deep neck infection; HALP, hemoglobin–albumin–lymphocyte–platelet; IQR, interquartile range; mHALP, modified HALP.

**Table 2:** Comparative receiver operating characteristic analysis of inflammatory and nutritional biomarkers for complicated deep neck infections.

Marker	AUC	95% CI	*p*-Value	Optimal Cut-Off	Sensitivity, %	Specificity, %
HALP	0.786	0.718–0.854	<0.001	≤2.7406	69.5	81.2
CRP	0.679	0.598–0.760	<0.001	≥175 mg/L	49.2	82.5
Albumin	0.692	0.607–0.777	<0.001	≤41 g/L	50.8	82.5
Lymphocyte count	0.719	0.643–0.795	<0.001	≤1.29 × 10^9^/L	50.8	83.1
mHALP	0.756	0.677–0.834	<0.001	≤0.0302	74.6	71.8

Footnote: Optimal cut-off values were determined using the Youden index. For HALP, albumin, lymphocyte count, and mHALP, lower values indicated a higher probability of complicated disease; for CRP, higher values indicated a higher probability. AUC, area under the receiver operating characteristic curve; CI, confidence interval; CRP, C-reactive protein; HALP, hemoglobin–albumin–lymphocyte–platelet; mHALP, modified HALP.

**Table 3:** Pairwise comparison of the mHALP receiver operating characteristic curve with HALP and individual laboratory markers.

Comparison	AUC Difference	95% CI	DeLong *p*-Value
mHALP vs. CRP	0.077	0.040–0.113	<0.001
mHALP vs. HALP	−0.030	−0.092–0.032	0.339
mHALP vs. albumin	0.064	−0.020–0.148	0.137
mHALP vs. lymphocyte count	0.037	−0.039–0.113	0.343

Footnote: AUC differences were calculated as AUCmHALP minus AUCcomparator. Positive values favor mHALP, whereas negative values favor the comparator. Correlated receiver operating characteristic curves were compared using the DeLong method. AUC, area under the receiver operating characteristic curve; CI, confidence interval; CRP, C-reactive protein; HALP, hemoglobin–albumin–lymphocyte–platelet; mHALP, modified HALP.

**Table 4:** Primary multivariable logistic regression analysis of factors associated with complicated deep neck infections.

Variable	Adjusted OR	95% CI	*p*-Value
Age, per 1-year increase	1.031	1.000–1.063	0.047
Maximum abscess diameter, per 1 mm increase	1.090	1.062–1.119	<0.001
Center 2 vs. Center 1	1.249	0.475–3.287	0.652
Log-transformed mHALP, per 1 SD increase	0.592	0.397–0.884	0.010

Footnote: The dependent variable was complicated deep neck infection. Center 1 was used as the reference category. Because of its right-skewed distribution, mHALP was natural-log-transformed and subsequently standardized. The adjusted odds ratio for mHALP therefore represents the change in the odds of complicated disease per one-standard-deviation increase in log-transformed mHALP. CI, confidence interval; mHALP, modified hemoglobin–albumin–lymphocyte–platelet score; OR, odds ratio; SD, standard deviation.

**Table 5:** Performance of biomarker-enhanced clinical models for complicated deep neck infections.

Model	Added Biomarker, Adjusted OR (95% CI); *p*-Value	Model AUC	AIC	Brier Score	LR *p*-Value
Clinical model	—	0.858	218.25	0.0829	—
Clinical model + HALP	0.458 (0.317–0.661); *p* < 0.001	0.894	201.37	0.0769	<0.001
Clinical model + CRP	1.054 (0.719–1.546); *p* = 0.786	0.858	220.17	0.0827	0.785
Clinical model + albumin	0.801 (0.576–1.115); *p* = 0.189	0.859	218.53	0.0822	0.191
Clinical model + lymphocyte count	0.449 (0.302–0.668); *p* < 0.001	0.883	202.05	0.0749	<0.001
Clinical model + mHALP	0.592 (0.397–0.884); *p* = 0.010	0.864	213.36	0.0794	0.009
Clinical model + HALP + CRP	HALP: 0.444 (0.304–0.650), *p* < 0.001; CRP: 0.876 (0.585–1.313), *p* = 0.522	0.896	202.97	0.0771	<0.001

Footnote: The clinical model included age, maximum abscess diameter, and study center. Biomarkers were added separately to the same clinical model, except for the model containing both HALP and CRP. HALP, CRP, lymphocyte count, and mHALP were natural-log-transformed and standardized; albumin was standardized without transformation. Adjusted odds ratios represent a one-standard-deviation increase in the corresponding biomarker. Likelihood-ratio *p*-values compare each nested biomarker-enhanced model with the clinical model and assess improvement in overall model fit. Pairwise changes in AUC relative to the clinical model were evaluated separately using the DeLong method and are reported in the Section 3. Lower AIC and Brier score values indicate better model performance. AIC, Akaike information criterion; AUC, area under the receiver operating characteristic curve; CI, confidence interval; CRP, C-reactive protein; HALP, hemoglobin–albumin–lymphocyte–platelet; LR, likelihood ratio; mHALP, modified HALP; OR, odds ratio. AUC and Brier score values in this table are apparent estimates; bootstrap-corrected estimates for the primary clinical plus mHALP model are reported in the Section 3. (—), not applicable; the clinical model served as the reference model for LR comparisons.

## Data Availability

The data presented in this study are available upon request from the corresponding author. The data are not publicly available due to privacy and ethical restrictions.

## Supplementary Materials

The following supporting information can be downloaded at https://www.mdpi.com/article/10.3390/diagnostics16162604/s1, Table S1: Secondary exploratory analysis of the ROC-derived mHALP cut-off for complicated deep neck infections.

## Abbreviations

The following abbreviations are used in this manuscript:

DNI(s)	Deep neck infection(s)
AIC	Akaike information criterion
CRP	C-reactive protein
IQR	Interquartile range
HALP	Hemoglobin–albumin–lymphocyte–platelet
mHALP	Modified hemoglobin–albumin–lymphocyte–platelet
AUC	Area under the curve
LR	Likelihood ratio
ROC	Receiver operating characteristic
CI	Confidence interval
OR	Odds ratio
