# Supplementary material for: Association of the Modified HALP Score with Disease Complexity in Deep Neck Infections: A Dual-Center Retrospective Study
Source: Diagnostics (Basel). 2026 Aug 17;16(16):2604. doi: 10.3390/diagnostics16162604 (PMC13512156; doi:10.3390/diagnostics16162604)
Supplement: Supplementary file 1 [file diagnostics-16-02604-s001.zip › diagnostics-4449534-supplementary.pdf]

Table S1. Secondary exploratory analysis of the ROC-derived mHALP cut-off for complicated deep neck infection.

**mHALP category**	**Non-complicated DNI (n=308)**	**Complicated DNI (n=59)**
>0.0302	221	15
≤0.0302	87	44
**Performance**		
**Measure**	**Result**	
Sensitivity	74.6%	
Specificity	71.8%	
Crude OR	7.451	
95% CI for crude OR	3.943–14.080	
Crude p-value	<0.001	
Adjusted OR	3.231	
95% CI for adjusted OR	1.491–7.002	
Adjusted p-value	0.003	

Footnote: The cut-off was selected using the Youden index in the same cohort in which its performance was evaluated and should therefore be regarded as exploratory. The adjusted model included age, maximum abscess diameter, and study center. CI, confidence interval; DNI, deep neck infection; mHALP, modified hemoglobin–albumin–lymphocyte–platelet score; OR, odds ratio; ROC, receiver operating characteristic.
